# Optical Biomarkers for the Diagnosis of Osteoarthritis through Raman Spectroscopy: Radiological and Biochemical Validation Using Ex Vivo Human Cartilage Samples

**DOI:** 10.3390/diagnostics11030546

**Published:** 2021-03-18

**Authors:** Paula Casal-Beiroa, Vanesa Balboa-Barreiro, Natividad Oreiro, Sonia Pértega-Díaz, Francisco J. Blanco, Joana Magalhães

**Affiliations:** 1 Unidad de Medicina Regenerativa, Grupo de Investigación de Reumatología (GIR), Instituto de Investigación Biomédica de A Coruña (INIBIC), Complexo Hospitalario Universitario de A Coruña (CHUAC), SERGAS, Universidade da Coruña (UDC), C/As Xubias de Arriba 84, 15006 A Coruña, Spain; paula.casalb@udc.es (P.C.-B.); natividad.oreiro.villar@sergas.es (N.O.); 2 Centro de Investigaciones Científicas Avanzadas (CICA), Universidade da Coruña (UDC), As Carballeiras S/N, Campus de Elviña, 15071 A Coruña, Spain; 3 Unidad de Epidemiología Clínica e Investigación Bioestadística, Instituto de Investigación Biomédica de A Coruña (INIBIC), Complexo Hospitalario Universitario de A Coruña (CHUAC), SERGAS, Universidade da Coruña (UDC), C/As Xubias de Arriba 84, 15006 A Coruña, Spain; vanesa.balboa.barreiro@sergas.es (V.B.-B.); sonia.pertega.diaz@sergas.es (S.P.-D.); 4 Centro de Investigación Biomédica en Red de Bioingeniería, Biomateriales y Nanomedicina (CIBER-BBN), Avenida Monforte de Lemos, 3-5, Pabellón 11, 28029 Madrid, Spain; 5 Grupo de Investigación de Reumatología y Salud (GIR-S), Departamento de Fisioterapia, Medicina y Ciencias Biomédicas, Facultad de Fisioterapia, Universidade da Coruña (UDC), Campus de Oza, 15008 A Coruña, Spain

**Keywords:** optical biomarkers, osteoarthritis, K-L grade, glycosaminoglycans, total collagen, lipids, calcium crystals

## Abstract

Osteoarthritis (OA) is the most common rheumatic disease, characterized by progressive articular cartilage degradation. Raman spectroscopy (RS) has been recently proposed as a label-free tool to detect molecular changes in musculoskeletal tissues. We used cartilage samples derived from human femoral heads to perform an *ex vivo* study of different Raman signals and ratios, related to major and minor molecular components of articular cartilage, hereby proposed as candidate optical biomarkers for OA. Validation was performed against the radiological Kellgren–Lawrence (K-L) grading system, as a gold standard, and cross-validated against sulfated glycosaminoglycans (sGAGs) and total collagens (*Hyp*) biochemical contents. Our results showed a significant decrease in sGAGs (SGAGs, A1063 cm^−1^/A1004 cm^−1^) and proteoglycans (PGs, A1375 cm^−1^/A1004 cm^−1^) and a significant increase in collagen disorganization (ColD/F, A1245 cm^−1^/A1270 cm^−1^), with OA severity. These were correlated with sGAGs or Hyp contents, respectively. Moreover, the SGAGs/HA ratio (A1063 cm^−1^/A960 cm^−1^), representing a functional matrix, rich in proteoglycans, to a mineralized matrix-hydroxyapatite (HA), was significantly lower in OA cartilage (K-L I vs. III–IV, *p* < 0.05), whilst the mineralized to collagenous matrix ratio (HA/Col, A960 cm^−1^/A920 cm^−1^) increased, being correlated with K-L. OA samples showed signs of tissue mineralization, supported by the presence of calcium crystals-related signals, such as phosphate, carbonate, and calcium pyrophosphate dihydrate (MGP, A960 cm^−1^/A1004 cm^−1^, MGC, A1070 cm^−1^/A1004 cm^−1^ and A1050 cm^−1^/A1004 cm^−1^). Finally, we observed an increase in lipids ratio (IL, A1450 cm^−1^/A1670 cm^−1^) with OA severity. As a conclusion, we have described the molecular fingerprint of hip cartilage, validating a panel of optical biomarkers and the potential of RS as a complementary diagnostic tool for OA.

## 1. Introduction

Osteoarthritis (OA) is a multifactorial disease that affects movable joints and one of the main causes of disability worldwide. It manifests as a set of molecular imbalances followed by physiologic and anatomical alterations of the joint tissues, such as articular cartilage extracellular matrix (ECM) degradation, subchondral sclerosis, and inflammation of the synovial membrane, which culminate in illness [1,2,3].

In its early stages, OA is asymptomatic, which hinders its prompt clinical diagnosis. In order to clinically assess a patient, pain and mobility questionnaires are performed, followed by radiography, and in some cases magnetic resonance imaging (MRI), which allows classification according to OA severity, under different scoring systems [4]. Even though these imaging techniques are routinely used for OA diagnosis, they present several disadvantages. On one hand, X-ray analysis is based on the observation of adjacent bone surface irregularities and remodeling, which limits its scope from moderate to advanced OA. On the other, MRI’s high cost and incompatibility for some patients, limits its application as a regular medical care practice [5]. Together with other unmet needs for successful diagnosis and treatment, these have spurred the investigation on biomarkers discovery and validation [6].

Raman spectroscopy (RS) is an optical technique that allows obtaining compositional information at the molecular level, being a non-invasive and label-free method [7]. It is based on the inelastic scattering that occurs when a monochromatic light interacts with the tissue sample analyzed, producing energy variations between the incident radiation with respect to the reflected. This Raman scattering is characteristic and specific of a vibrational mode, bond, or molecule present in the tissue composition, producing an unique molecular fingerprint [8]. Typical Raman spectra are displayed at different intensities with corresponding wavelength shifts (cm^−1^) specific to molecular vibrations. Thereafter, raw data is processed and can be classified according to unsupervised or supervised statistical methods, allowing sample classification and diagnostic analysis [7].

Recently, RS has been explored in different rheumatologic diseases, including OA, covering different joint tissues, including articular cartilage, synovium, bone, and to a less extent, the meniscus, tendons, and ligaments. For each individual tissue, a set of optical biomarker candidates for OA has been proposed, further supporting RS potential as a diagnostic method [9,10,11,12,13,14].

Since the first analysis of human articular cartilage by RS [15], several authors have reported differences found in specific Raman peaks or ratios related to major ECM components that can be associated to molecular events that occur during OA progression. Such changes can be summarized in alterations on the relative distribution of the collagen secondary structure (amide III, C-N stretching, random coil and α-helix, at 1245 and 1270 cm^−1^, respectively) [16,17,18], and the decrease of sulphated glycosaminoglycans (GAGs) content (OSO_3_^−^ symmetric stretching, at 1063 cm^−1^) [15,18]. Additionally, studies reported the presence of phosphate and carbonate hydroxyapatite peaks (~958 cm^−1^ and ~1070 cm^−1^, respectively) in injured cartilage and hypertrophic zones [15,19]. The occurrence of tissue mineralization and remodeling processes have also been associated with the presence of the aforementioned Raman peaks, in damaged tissues, on an OA rat model [20].

So far, RS studies on OA cartilage have covered different anatomical areas of the joint (tibial plateau and femoral condyle) [17,18,21] but few have referenced histopathological scoring systems (e.g., the Collins pathological scale) [17]; even on the cellular level, only one study used chondrocytes isolated from different OA stages, based on the International Cartilage Repair Society (ICRS) system [18]. In overall, sample sizes used are limited and there is a lack of validation against clinical gold standards or cross-validation against biochemical tools [17,22].

In this work, we propose to study the molecular alterations on human cartilage derived from the femoral heads of a cohort of forty-seven healthy and radiological OA patients, cross-validated against biochemical assays for sulfated-GAGs (sGAGs) and total collagen (*Hyp*). Raman spectroscopy is further proposed as a complementary diagnostic tool for OA, through the validation of optical biomarkers.

## 2. Materials and Methods

### 2.1. Study Cohort and Sample Collection

Human articular cartilage samples (plugs of 6 mm diameter and ~1 to 1.5 mm thick) were extracted from the femoral head of 47 donors, 24–48 h, after joint replacement intervention, frozen in liquid nitrogen and stored at −80 °C, until further processing. The inclusion factor was samples from patients with available index femoral head radiography for the assessment of OA severity, by clinical doctors, according to the Kellgren-Lawrence (K-L) grading scale as follows: grade 0: no pathological features; grade I: doubtful narrowing of joint space and possible osteophytic lipping; grade II: definite osteophytes and possible narrowing of joint space; grade III: moderate multiple osteophytes, definite narrowing of joint space, some sclerosis, and possible deformity of bony ends, and grade IV: large osteophytes, marked narrowing of joint space, severe sclerosis, and definite deformity of bone ends [5]. Six donors were thus classified as healthy (K-L = 0) and forty-one as OA (K-L ≥ I–IV). The final cohort (Table 1) was composed of cartilage samples from donors aged between 42 and 94 years (mean age of 72), with a female/male ratio close to 3:2.

### 2.2. Raman Spectra Obtainment and Data Processing

Raman spectra were collected from the surface of cartilage samples using a WITec Alpha300R+ Raman spectrometer (WITec focus innovations, Ulm, Germany), equipped with a Near-Infrared (NIR) laser (λ = 785 nm) with incident laser radiation of 50 mW, 120 scans, 4 cm^−1^ resolution and an acquisition time of 1 s, to achieve a good signal-to-noise ratio. Once spectra were acquired, baseline correction and analysis were carried out using MagicPlot version 2.7 software (Magic Plot Systems, LLC). Briefly, linear baseline correction was performed by limiting the selected wavenumber intervals, related with the biochemical vibrations of interest. Peaks’ assignment was then performed based on available literature. For a quantitative analysis, the main peaks were integrated based on a Gaussian distribution, adjusting the areas’ sum to the spectral profile in order to predict signals’ overlaps. An example of the area measurement in an arbitrary spectrum is presented as supplementary material (Figure S1). Proposed optical biomarkers (Table 2) were calculated based on the peaks’ area values, and a relative quantification was performed using phenylalanine (Phe) (1004 cm^−1^), as normalization peak [15,16,18,19].

### 2.3. sGAGs and Total Collagen Biochemical Content Analysis

After RS analysis, cartilage samples were frozen at −80 °C and lyophilized in a Telstar Cryodos (Telstar, Barcelona, Spain), at −0.1 mbar, for 18 h. Explants (10–50 mg) were digested with papain (2.5 × 10^−2^ mg/mL) in 0.2 M sodium phosphate buffer (Na_2_HPO_4_^−^NaH_2_PO_4_, pH 6.4), 8 mg/mL sodium acetate, 4 mg/mL EDTA and 0.8 mg/mL L-cysteine-HCl (all from Sigma-Aldrich, Darmstadt, Germany), at 65 °C, for 18 h. The amount of sulfated GAGs (sGAGs) was quantified using the dimethyl methylene blue (DMMB) dye-binding assay (Blyscan, Biocolor Ltd., Carrickfergus, UK) with a chondroitin sulphate standard and normalized against the explants’ wet weight (w.wt). For the quantification of total collagen, an acid hydrolysis of the digested supernatant was performed, by adding HCl 37% (Panreac AppliChem, Darmstadt, Germany) at a ratio of 1:1, in Teflon-sealed borosilicate glass vials (Lab Logistics Group, Meckenheim, Germany), at 120 °C, for 18 h. The total collagen content was then measured using the hydroxyproline (*Hyp*) assay kit (Sigma-Aldrich, Darmstadt, Germany) with hydroxyproline standards and normalized against each sample’ w.wt. Both sGAGs and Hyp absorbance readings were carried out for each cartilage sample in duplicate, in 96-well plates, at 656 nm or 560 nm, respectively, using a Sinergy HTX reader (BioTek, Winooski, VT, USA).

### 2.4. Statistical Analysis

All statistical analyses were carried out using R statistical open software (version R 3.5.1). Kruskal–Wallis non-parametric tests with Bonferroni correction were performed. Spearman’s correlation coefficients *rho* were determined in order to compare the proposed biomarkers versus radiological K-L grade or biochemical parameters, sGAGs and Hyp. All values are reported ± as means standard deviation (SD). Significance was accepted at a level of *p* < 0.05.

## 3. Results

### 3.1. Molecular Alterations during Radiological OA Progression—Raman Spectra Analysis

**Figure 1:**
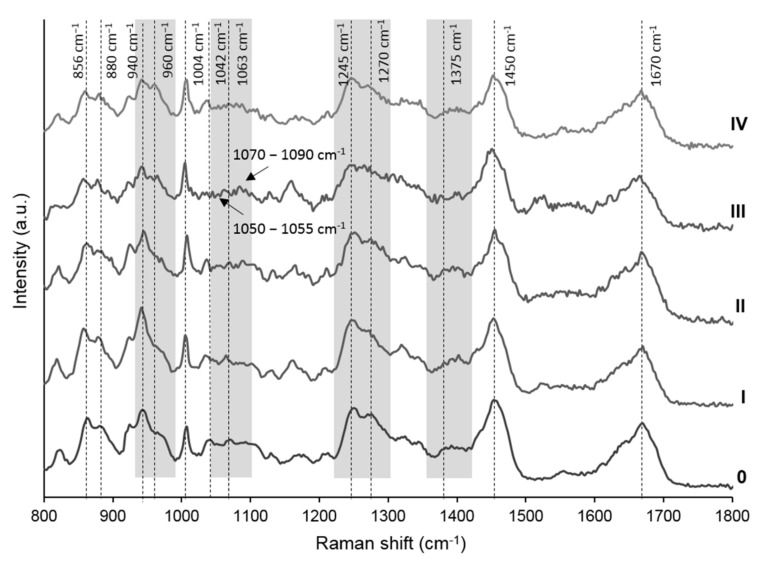
Raman spectra obtained from cartilage explants derived from the femoral heads of patients with different Kellgren-Lawrence (K-L) radiological OA severity grades (0–IV), obtained at a λ = 785 nm with incident laser radiation of 50 mW, 120 scans, 4 cm^−1^ resolution and 1 s acquisition time.

Raman spectra in the 800–1800 cm^−1^ region, of human cartilage explants, derived from healthy donors (K-L 0) and patients with different radiological OA grading (K-L I to IV) were depicted in Figure 1.

The main peaks identified, summarized in Table 3, were those obtained at Raman shifts as follows: 850–880 cm^−1^, corresponding to the C–C bond stretching of proline (Pro) and hydroxyproline (Hyp) [16,23]; 920–928 cm^−1^, corresponding to the C–C stretching of proline [16]; 940 cm^−1^, corresponding to both the symmetric stretching mode of the O–glycosidic bond and the protein C–C stretching [19,23,24]; 1004 cm^−1^, corresponding to the aromatic ring stretching of phenylalanine (Phe) [15]; 1042 cm^−1^, assigned to the C–O–C stretching, of the pyranose ring of GAGs [23]; 1063 cm^−1^, assigned to the symmetric stretching of O–SO_3_^−^ of sulfated GAGs [15,18,19,23]; 1245–1270 cm^−1^ doublet, corresponding to the C–N stretching of amide III, random coil or α-helix structure of collagen, respectively [17]; 1375 cm^−1^, corresponding to the symmetric stretching of the methyl (–CH_3_) group, related to proteoglycans (PGs) [21]; ~1450 cm^−1^, corresponding to the vibrational deformation modes (CH_2_), from lipids and proteins, and ~1670 cm^−1^, assigned to C=O stretching of amide I, present in collagen and other proteins [15,16,19].

**Figure 2:**
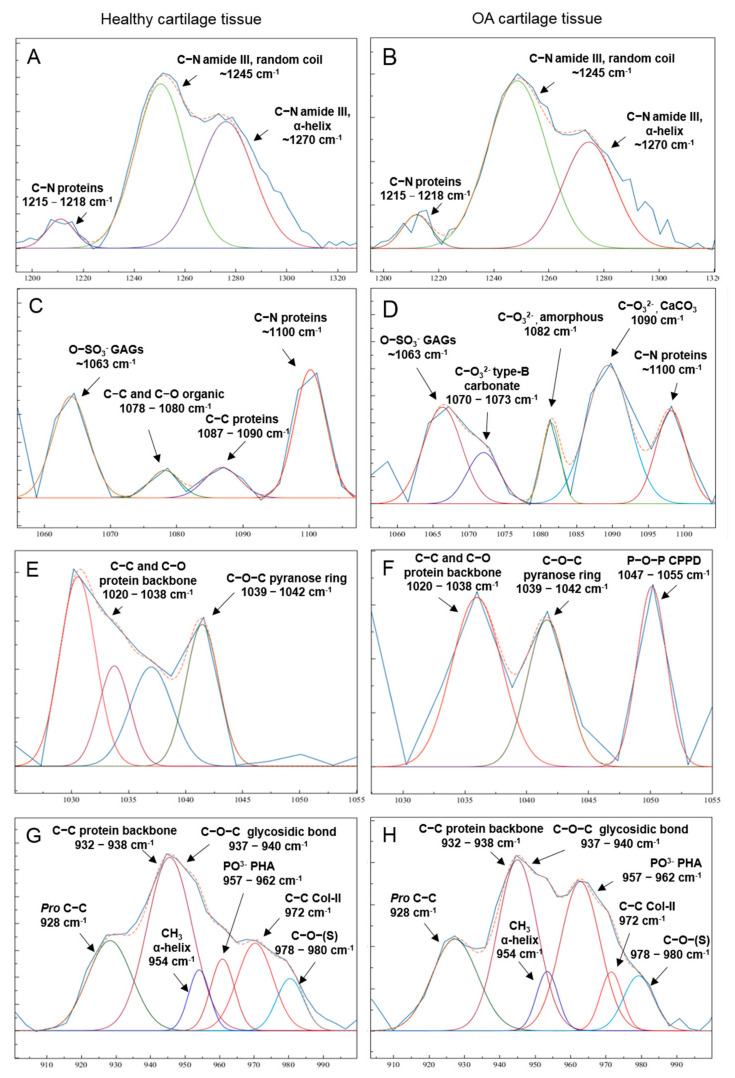
Deconvolution of peaks of the principal molecular alterations observed in healthy (K-L 0, left column) and radiological OA (K-L IV, right column) cartilage. Regions of interest (ROI) are depicted as follows, (**A**,**B**) 1200–1300 cm^−1^; (**C**,**D**) 1060–1100 cm^−1^; (**E**,**F**) 1020–1055 cm^−1^; (**G**,**H**) 900–1000 cm^−1^.

Several differences between healthy and OA cartilage Raman spectra were observed. For a better comprehension, Figure 2 shows different regions of interest (ROI) and corresponding peaks deconvolution. Grossly, concerning collagen, it was observed a decrease in the 1270 cm^−1^ peak, related to a α-helix secondary structure, with respect to ~1245 cm^−1^, related to collagen’s random coil configuration (Figure 2A,B) [17,18]. A decrease in peaks related to PGs (1063 and 1375 cm^−1^) [21], in severely damaged cartilage (K-L IV) was observed (Figure 2C,D).

Moreover, the appearance or increase of peaks related to mineralization was observed in OA cartilage. Different types of inorganic components were found, such as carbonate compounds in the 1060–1100 cm^−1^ region (Figure 2C,D, Table 3) and calcium pyrophosphate dehydrate deposits (CPP), in the 1020–1055 cm^−1^ region (Figure 2E,F, Table 3) [27]. An overlap of the carbonated hydroxyapatite peak (type-B carbonate, 1070 cm^−1^) [21] with the 1063 cm^−1^ peak, correspondent to sulfate groups could be observed. Other carbonate related peaks detected, in OA cartilage (Figure 2D), could be related with amorphous carbonate (~1080 cm^−1^) and crystalline carbonate compounds, as calcium carbonate (~1090 cm^−1^) [15,21,25,29,30,31]. Moreover, an increase of the ~960 cm^−1^ peak, from hydroxyapatite phosphate groups, can be associated with tissue mineralization during OA progression (Figure 2G,H) [18,19,25,26].

### 3.2. Molecular Alterations during OA Progression—K-L Validation

**Figure 3:**
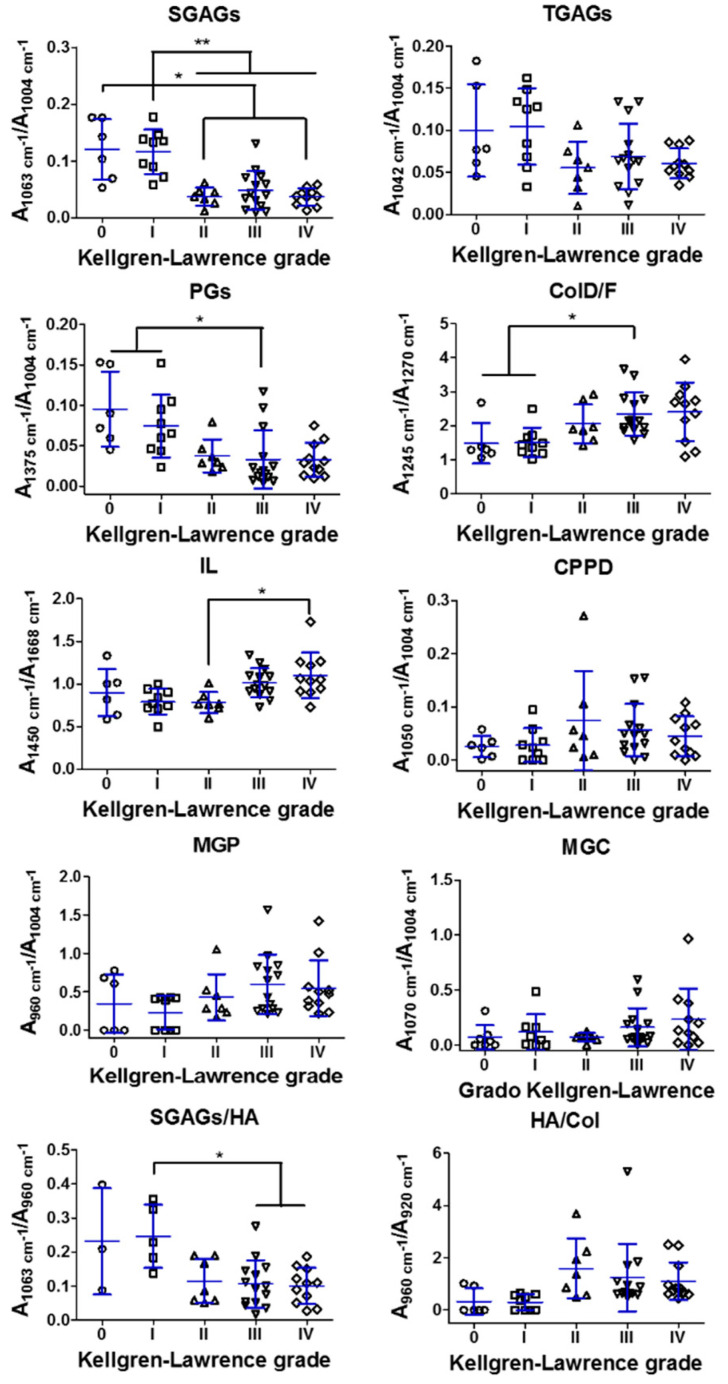
Quantitative results of Raman ratios related to cartilage components, glycosaminoglycans (SGAGs, TGAGs, PGs), collagen (ColD/F), lipids (IL) and tissue mineralization (directly related: CPPD, MGP, MGC and indirectly related: SGAGs/HA and HA/Col) of cartilage, derived from the femoral head, of healthy (K-L 0) and radiological OA (K-L I–IV) patients. Values are mean ± SD. Symbols (∆, □, ◦, ◊) represent individual samples. Significance is indicated as * *p* < 0.05 and ** *p* < 0.01.

Ratios related to glycosaminoglycans, i.e., “sulphated GAGs” (SGAGs, A1063 cm^−1^/A1004 cm^−1^), “total GAGs” (TGAGs, A1042 cm^−1^/A1004 cm^−1^) and “PGs” (A1375 cm^−1^/A1004 cm^−1^), showed a decrease with increasing K-L grade (Figure 3). Among these parameters, only SGAGs and PGs showed statistically significant differences, detected between healthy and doubtful OA (K-L 0–I grades) with mild to severe OA (K-L II–IV grades) as follows, K-L 0 vs. K-L II and IV (*p* < 0.05) and K-L I vs. K-L II–IV (*p* < 0.01), K-L 0–I vs. K-L III (*p* < 0.05), respectively. These results were supported by negative correlations obtained for SGAGs (*rho* = −0.632, *p* < 0.001), PGs (*rho* = −0.532, *p* < 0.001) and TGAGs (*rho* = −0.324, *p* = 0.025) (Table 4).

Regarding the parameter related to the configuration of collagen fibers, “Defective/Functional Collagen” ratio (ColD/F, A1245 cm^−1^/A1270 cm^−1^) increased with OA severity, obtaining significant differences between K-L 0–I vs. K-L III (*p* < 0.05) (Figure 3), supported by a positive correlation (*rho* = 0.529, *p* < 0.001) (Table 4).

The “Indirect Lipid index” ratio (IL, A1450 cm^−1^/A1668 cm^−1^) showed an increasing trend, with significant differences found only between mild and severe radiological OA (K-L II vs. IV, *p* < 0.05) (Figure 3) and a correlation with K-L (*rho* = 0.427, *p* = 0.002) (Table 4).

For parameters directly related with tissue mineralization, i.e., “Mineralization—Phosphate Groups” (MGP, A960 cm^−1^/A1004 cm^−1^), “Mineralization—Carbonate Groups” (MGC, A1070 cm^−1^/A1004 cm^−1^), “calcium pyrophosphate dihydrate deposits” (CPPD, A1050 cm^−1^/A1004 cm^−1^), we found an increasing trend, in regard with OA severity, for both MGP and MGC parameters, whilst CPPD presented higher variability (Figure 3). A weak correlation was found for MGC (*rho* = 0.293, *p* = 0.043) (Table 4).

On the other hand, for indirectly related mineralization parameters, an increasing trend with OA severity was found for “Phosphate Hydroxyapatite/Collagen” ratio (HA/Col, A960 cm^−1^/A920 cm^−1^) (Figure 3), indicative of the relative amount of mineralized tissue in regard to a collagenous matrix [15], supported by a positive correlation (*rho* = 0.446, *p* = 0.001) (Table 4). An opposite behavior was found in the SGAGs/HA ratio (A1063 cm^−1^/A960 cm^−1^), indicative of the relative amount of functional extracellular matrix, rich in GAGs, with respect to the mineralized matrix [15], with significant differences between K-L I and K-L III–IV (*p* < 0.05) (Figure 3) and a negative correlation (*rho* = −0.426, *p* = 0.005) (Table 4).

### 3.3. Molecular Alterations during OA Progression—Biochemical Cross-Validation

**Figure 4:**
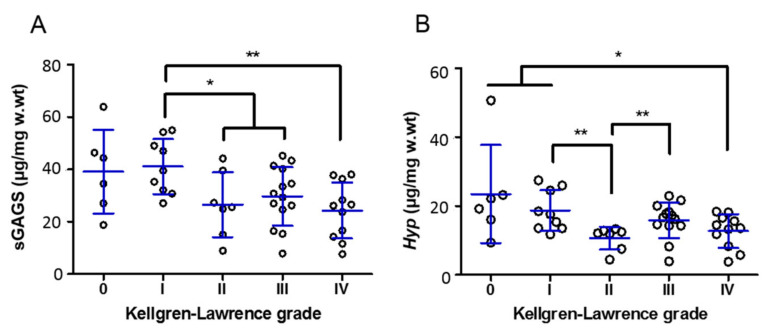
Biochemical content of sGAGs (**A**) and total collagens (*Hyp*) (**B**) of articular cartilage with different Kellgren–Lawrence (K-L) grades. Values are mean ± SD. Dots represent individual samples. Significance is indicated as * *p* < 0.05 and ** *p* < 0.01.

The sGAGs content obtained biochemically was observed to significantly decrease with the progression of cartilage degradation, from values of 41.10 ± 10.52 (K-L I) to 26.49 ± 12.42 (K-L II) and 29.69 ± 11.16 µg/mg w.wt (K-L III) (both *p* < 0.05) and 24.26 ± 10.68 µg/mg w.wt (K-L IV, *p* < 0.01) (Figure 4A). This finding is consistent with a decrease observed in the proposed Raman biomarkers related with GAGs and PGs (Figure 2).

**Figure 5:**
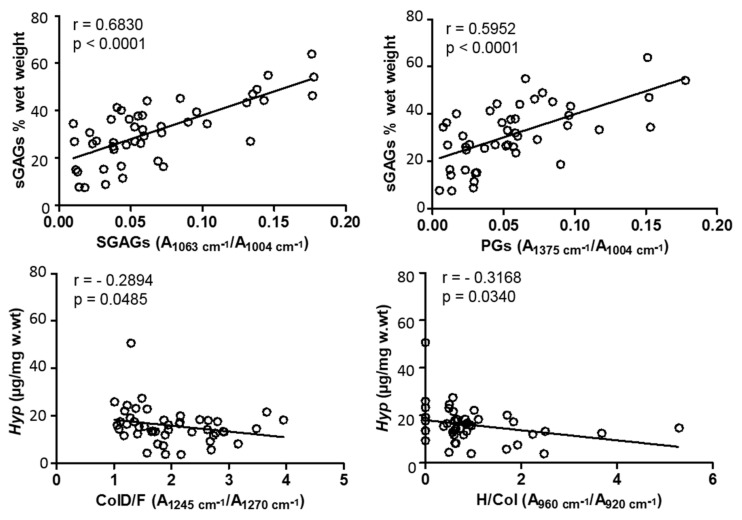
Correlations obtained between sGAGs and total collagens (*Hyp*) biochemical content and related optical biomarkers, SGAGs (A1063 cm^−1^/1004 cm^−1^) and PGs (A1375 cm^−1^/1004 cm^−1^), and ColD/F (A1425 cm^−1^/1270 cm^−1^) and H/Col (A960 cm^−1^/920 cm^−1^), respectively. Spearman’s *rho* coefficients and *p*-values are indicated. Significance was considered for *p* < 0.05.

Furthermore, sGAGs content was found to be positively correlated with the Raman parameters SGAGs (A1063 cm^−c^/1004 cm^−1^) and PGs (A1375 cm^−^/1004 cm^−1^), obtaining a higher value for SGAGs, which is directly related to sulphate groups (*rho* = 0.6830), when compared with PGs (*rho* = 0.5952) (both *p* < 0.0001) (Figure 5). No significant correlation was detected between sGAGs biochemical content and the TGAGs parameter (*rho* = 0.2659, *p* = 0.075) (data not shown).

Regarding total collagens content obtained biochemically, a significant decrease was observed with OA severity, namely, K-L 0 and I vs. K-L IV (*Hyp* = 23.47 ± 14.2 and 18.71 ± 5.81 vs. 12.71 ± 4.87 µg/mg w.wt, *p* < 0.05), K-L I vs. K-L II (*Hyp* = 18.71 ± 5.81 vs. 10.65 ± 3.33 µg/mg w.wt, *p* < 0.01) and K-L II vs. K-L III (*Hyp* = 10.65 ± 3.33 vs. 15.88 ± 1.43 µg/mg w.wt, *p* < 0.01) (Figure 4B). *Hyp* was found to be negatively correlated with Raman biomarkers ColD/F (A1245 cm^−1^/A12070 cm^−1^) and HA/Col (A960 cm^−1^/A920 cm^−1^), with rho = −0.2894 (*p* = 0.0485) and rho = −0.3168 (*p* = 0.0340), respectively (Figure 5). These results suggest that *Hyp* biochemical content was lower in cartilage samples where both a greater relative disorganization of collagen and bone to collagenous matrix ratio were observed, in the course of OA severity (Figure 2 ColD/F and HA/Col, respectively).

## 4. Discussion

OA is a complex and multifactorial disease characterized by major changes that succinctly consist on the disorganization of the articular cartilage, edema, chondrocyte apoptosis, tissue loss, and subchondral bone changes [2]. Even though structural, cellular, and molecular processes during cartilage degradation have been thoroughly investigated, OA pathogenesis is far from being understood. As such, early diagnosis, biomarkers validation and personalized treatments are still some of its major unmet needs [32,33].

Raman spectroscopy has been previously used to describe the molecular fingerprint of articular cartilage derived from different joint tissues [15,16,18,19,23]. However, there is still a lack of consistent studies that can validate the use of Raman signals or derived ratios for the detection and follow-up of molecular changes in the onset and progression of OA. For this study, focused on hip OA, we have selected and proposed an optical biomarker panel, based on a previous literature review, considering representative parameters, related with major and minor cartilage ECM components [13].

From the parameters related with GAGs, we demonstrated negative correlations between SGAGs and PGs with radiological OA severity. These were both cross-validated against sGAGs biochemical content, which support the decrease in proteoglycans that occurs in OA cartilage [34]. SGAGs ratio hereby proposed as a candidate biomarker was previously studied by Kumar et al., obtaining similar results regarding the ICRS grading system [18]. It is worth noting that, from all the candidate biomarkers, SGAGs was the only where a significant difference was found between healthy–doubtful OA and moderate radiological grade (K-L 0–I vs. II) (Figure 3). The lack of differences between healthy cartilage samples and those in early stages of OA may be due to compensatory synthesis mechanisms of chondrocytes in response to initial damage [34]. Moreover, SGAGs, being directly related to sulphate groups, has potential in assessing the sulphation loss in the cartilaginous tissue during its degradation [35]. Water content was also found increased, although this was not significant (data not shown).

Considering other major component of cartilage ECM, we have studied the ColD/F parameter, proposed by other authors, to characterize the disorganization of the relative structure of collagen [17,18]. This parameter that significantly increased with OA severity, also correlated with K-L grade. The observed increase in the random coil collagen structure signal (i.e., defective collagen), could be indicative of denaturation or metabolic changes in the collagen synthesis process. OA chondrocytes have been described to synthetize type-X collagen in hypertrophic cartilage and type-VI collagen in interterritorial zones [34,36]. These two types of collagen have large non-helical terminal domains, and considerably smaller α-helix domains than those of type-II collagen (460 and 200 amino acids respectively, compared to 1000 amino acids) [37,38]. Therefore, the increase in the ColD/F parameter could be due to the synthesis of both type-X and -VI collagens.

Although previous studies showed total collagens content to be either increased or not altered during OA, by synthesis/degradation processes [39,40,41,42], in our case, *Hyp* biochemical content significantly decreased with OA severity, which could be related to the stability of type-II collagen triple helix structure domains [43]. Furthermore, the association of this amino acid with the stability of the collagen structure was confirmed, finding a weak, but significant correlation, indicating a greater relative disorganization of the collagen fibers (higher ColD/F values) with a decrease in Hyp residues.

HA/Col showed a negative correlation with *Hyp* content, indicating a lower relative collagen content with an increasing mineralization, which could be related to nucleation and crystal deposition processes around collagen fibers. The mineralization process could also be favored by a decrease in proteoglycans and sGAGs in cartilage ECM during OA progression [44,45], supported in our case, by a decrease in the SGAGs/HA parameter.

Mineralization and tissue remodeling are processes that occur during OA progression. Basic calcium phosphate (BCP), such as hydroxyapatite (HA), and calcium pyrophosphate dihydrate (CPP) are commonly deposited in OA and aging cartilage and can directly promote the production of catabolic and pro-inflammatory cytokines and enzymes by chondrocytes and synovial cells [46]. The pathogenic mechanism of calcium crystals deposition is still not fully elucidated and calcium-containing crystal deposition diseases are commonly underdiagnosed.

Even though both crystal types have been shown to coexist in cartilage [47], other authors have rarely found them in the same articular tissue (condyle vs. tibial plateau) or zonal distribution (lateral vs. medial compartments) [44]. In our study, we detected the concomitant presence of spectral bands for molecular functional groups associated with both BCP (MGP and MGC) and CPPD, in 68% of the samples (data not shown). Even though CPPD was present in the majority of samples (87.5%) (data not shown), its deposition was not correlated with OA severity.

Indeed, others have demonstrated that in some instances CPPD in cartilage middle zone can accumulate in the absence of cartilage lesions. Although the same authors also reported superficial zone crystal deposition to be linked with cartilage lesions, suggesting an intimate association between metabolically altered and fissured cartilage with crystal nucleation that worsens the tissue mechanical damage [48].

The relatively unique capacity of chondrocytes to produce extracellular inorganic pyrophosphate is linked to the promotion of chondrocalcinosis [49]. In our case, three cases of chondrocalcinosis were confirmed during radiography examination, from which, two samples had the highest CPPD (K-L I) or MGC (K-L III) ratios (Figure 3), although these could also be related to other degradation processes associated with age [50].

On the other hand, only HA-related signals (phosphate—MGP and carbonate—MGC) showed an increasing trend with OA severity, supported by a significant correlation for the latest. A relation between BCP crystals presence in the synovial fluid of patients with radiologic knee OA has previously been shown [51]. BCP crystals have also been associated with secondary forms of arthropathies or calcific periarthritis [46]. In our cohort, we found one sample, that concomitantly presented one of the highest MGC and CPPD ratios (K-L IV, Figure 3), to be a diagnosed case of psoriatic arthritis. These results support further investigation on the potential of RS in the diagnosis of calcium crystal deposition musculoskeletal diseases, complemented with other techniques, such as histology and polarized light microscopy, in order to elucidate the contribution of crystals deposition in cartilage degradation and remodeling processes [52].

Finally, when considering ECM minor components, such as lipids, this is the first study that reports IL for cartilage molecular characterization, based on Raman peaks previously described for other tissues [53,54]. We observed a significant increase of the IL parameter, for moderate and severe radiological hip OA samples, supported by a positive correlation. These results could be related to an accumulation of lipids in OA cartilage and concomitant protein low abundance due to ECM proteolysis, as described by mass spectrometry [55,56]. Others have shown in cellular studies using chondrocytes an increase in lipid deposition, by considering other Raman signals attributed to lipids (~1304 cm^−1^) with the advancement of OA [28]. Nonetheless, more studies are needed to elucidate the role of lipid deposition in OA progression. Other variable to have into account is body mass index (BMI), which has been shown to influence on the quantification of the peak ~1450 cm^−1^ [54]. Being obesity a risk factor for OA, BMI values could be considered in future analyses. Unfortunately, these data were not available for the current study.

Even though this study sheds light in the potential of optical biomarkers for OA diagnosis, it presents some limitations. This study constitutes the biggest cohort on hip-OA with RS to date, although due to lack of access to radiographies from samples derived from healthy tissue, only six samples were available. Moreover, some of these samples, even though found no radiological signs of OA, already presented some macroscopically identified lesions. In contrast and due to access derived from replacement surgeries, an over-representation of K-L IV grade samples was obtained.

Another limitation was that cartilage samples were not always obtained from the same compartment of the hip and thus our cohort comprises samples from both load and non-load areas that have been associated with different RS profiles [17]. Nevertheless, we conducted a comparison between RS parameters in cartilage derived from lesion and adjacent sites and we could only find significant differences for SGAGs and HA/Col in K-L II samples (data not shown), which can be an indicative that molecular alterations could already be predicted in adjacent and macroscopically intact cartilage. This finding is noteworthy when considering future translation of RS for OA diagnosis in clinical settings, already being tested in vivo under laser coupling with fiber optic probe systems [15,57,58].

In summary, described findings in this study support the association of the different optical biomarker candidates with OA-cartilage molecular changes that include, a decrease in proteoglycans that can be related to the action of aggrecanases and metalloproteases (MMP) and a lower expression of aggrecan by chondrocytes [34,59], sulphation loss [40], alterations or denaturation of the collagen network [39,60], and nucleation of calcium crystals around altered collagen fibers [45,61].

## 5. Conclusions

We have successfully described the molecular fingerprint of human articular cartilage derived from the femoral head of healthy and radiological OA patients. Our results support SGAGs, PGs, and ColD/F as optical OA biomarker candidates, obtaining significant correlations with K-L grade as gold standard and cross-validated against sGAGs and *Hyp* biochemical contents. IL and calcium-related signals (MGP, MCP, and CPPD) have also shown potential and need further validation for their role in elucidating tissue remodeling and mineralization processes during OA. Finally, this study supports Raman spectroscopy application as a diagnostic complementary tool for hip OA.

## Figures and Tables

**Table 1:** Demographics and clinical diagnostic of the patients included in this study.

**Demographics**			
Sex	Female (F)	*n* = 27	57.4%
	Male (M)	*n* = 20	42.6%
Age	Range	42–94 years old	
	Mean ± SD	72 ± 12 years old	
**Diagnostic**			
Radiological grade (K-L)	Healthy (K-L 0)	*n* = 6	
	OA (K-L I–IV)	*n* = 41	

**Table 2:** Optical biomarker candidates for osteoarthritis (OA) in human cartilage derived from the femoral head.

Optical Biomarker	Molecular Component or Associated Event	Acronym	Definition
A960 cm^−1^/A1004 cm^−1^	Mineralization—Phosphate Groups	MGP	Phosphate groups present at hydroxyapatite
A960 cm^−1^/A920 cm^−1^	Phosphate Hydroxyapatite/Collagen	HA/Col	Bone to collagen
A1039–42 cm^−1^/A1004 cm^−1^	Total GAGs	TGAGs	Total glycosaminoglycans
A1050 cm^−1^/A1004 cm^−1^	CPPD	CPPD	Calcium pyrophosphate dihydrate deposits
A1063 cm^−1^/A1004 cm^−1^	Sulphated GAGs	SGAGs	Sulphated glycosaminoglycans (OSO_3_^−^ groups)
A1063 cm^−1^/A960 cm^−1^	Sulphated GAGs/Phosphate	SGAGs/HA	Cartilage to bone
A1070 cm^−1^/A1004 cm^−1^	Mineralization—Carbonate Groups	MGC	Mineralization grade-carbonated hydroxyapatite
A1245 cm^−1^/A1270 cm^−1^	Defective/Functional Collagen	ColD/F	Collagen randomness—as the relative amount of collagen random coil (defective Col) to an α-helix structure (functional Col)
A1375 cm^−1^/A1004 cm^−1^	Proteoglycans	PGs	Proteoglycans
A1450 cm^−1^/A1668 cm^−1^	Indirect Lipid Index	IL	Relative amount of unspecific lipids and proteins to the total protein content

1004 cm^−1^, Phenylalanine (Phe) peak used for normalization.

**Table 3:** Raman peaks assignments of articular cartilage molecular fingerprint, obtained from healthy and radiological OA tissues.

Raman Shift (cm^−1^)	Assigned Bond/ Molecule	Component	References
850–880 856–858 875–880	C–C stretching *Pro* *Hyp*	Collagen	[16,23]
920–928	C–C stretching *Pro*	Collagen	[15,16]
932–941 932–938 937–941	Symmetric stretching: C–C protein backbone C–O–C α 1–4 glycosidic bond	Collagen GAGs	[16,19,23,24]
954–962	PO_4_^3−^, symmetric stretching	Phosphate hydroxyapatite (HA)	[15,19,23,25,26]
1004	Aromatic ring stretching phenylalanine (*Phe*)	Proteins	[15,16,19]
1039–1042	C–O–C stretching pyranose ring	GAGs	[23]
1047–1055	P–O–P symmetric stretching	CPPD	[27]
1060–1064	O–SO_3_^−^ symmetric stretching	Sulphated GAGs, PGs	[15,19,21,23,28]
1070–1090 1070–1073 1080–1082 1090	CO_3_^2−^, asymmetric stretching Type-B carbonate Amorphous carbonate CaCO_3_	Carbonate Carbonated hydroxyapatite Amorphous carbonate Calcium carbonate deposits	[15,21,24,25,29]
1230–1280 1245 1270	C–N stretching amide III: random coil α-helix structure	Collagen: Defective Functional	[16,17,18,21,23]
1375–1380	CH_3_ symmetric stretching	GAGs, PGs	[21]
1441–1460	CH_2_ deformation/scissoring	Protein and lipids	[15,16]
1630–1690 1645–1655 1660–1670 1665–1675	C=O stretching amide I: α-helix structure random coil β-sheet structure	Collagen and other proteins	[15,16,19]
1550–1600	N–H and C–N deformation amide II	Proteins	[24]

**Table 4:** Spearman’s *rho* coefficients and *p*-values for proposed Raman biomarkers and Kellgren–Lawrence (K-L) grading system. Significance was considered for *p* < 0.05.

		SGAGs	TGAGs	PGs	ColD/F	IL	CPPD	MGP	MGC	SGAGs/HA	HA/Col
	*rho*	−0.632	−0.324	−0.532	0.529	0.427	0.191	0.321	0.293	−0.426	0.446
	*p*	<0.001	0.025	<0.001	<0.001	0.002	0.219	0.260	0.043	0.005	0.001

## Supplementary Materials

The following are available online at https://www.mdpi.com/2075-4418/11/3/546/s1, Figure S1: Example of area measurement by MagicPlot software, in wavenumber range 1045–1080 cm^−1^, of a Raman spectrum from an arbitrary cartilage sample.
