# Supplementary material for: Optical Biomarkers for the Diagnosis of Osteoarthritis through Raman Spectroscopy: Radiological and Biochemical Validation Using Ex Vivo Human Cartilage Samples
Source: Diagnostics (Basel). 2021 Mar 18;11(3):546. doi: 10.3390/diagnostics11030546 (PMC8003208; doi:10.3390/diagnostics11030546)
Supplement: Supplementary file 1 [file diagnostics-11-00546-s001.pdf]

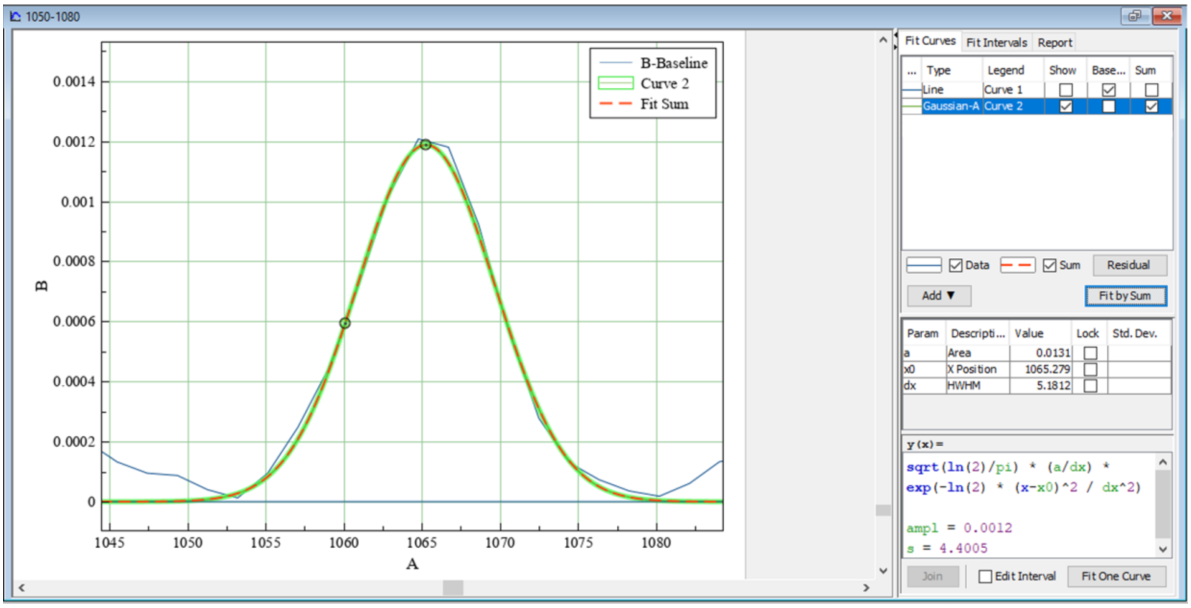

Figure S1. Example of area measurement by MagicPlot software, in wavenumber range 1045–1080 cm^{-1}, of a Raman spectrum from an arbitrary cartilage sample.
